# Prevalence and characteristics of prostate cancer among participants of a community-based screening in Nigeria using serum prostate specific antigen and digital rectal examination

**DOI:** 10.11604/pamj.2013.15.129.2489

**Published:** 2013-08-10

**Authors:** Stephen Odunayo Ikuerowo, Olufunmilade Adefolarin Omisanjo, Muftau Jimoh Bioku, Michael Olawale Ajala, Victor Patrick Nonyelim Mordi, Julius Olusanmi Esho

**Affiliations:** 1 Lagos State University College of Medicine, Ikeja, Lagos, Nigeria; 2 Lagos State Pathology Services, General Hospital, Lagos, Nigeria

**Keywords:** Prostate, cancer, prevalence, screening, Nigeria

## Abstract

**Introduction:**

Prostate cancer (CaP) is the most commonly diagnosed cancer among Nigerian men but CaP screening is not a common practice. The true burden of the disease in Nigeria is not known. The study was aimed at studying the community burden of CaP in Lagos.

**Methods:**

During a community-based prostate cancer awareness program in 13 local government areas of Lagos, men aged >40 years had serum total PSA (tPSA) test and digital rectal examination (DRE). Those with abnormal DRE or tPSA >95th percentile of the cohort or both were selected for prostate biopsy (TRPB).

**Results:**

4172 men were screened and complete data was available for 4110 (98.5%). The mean age was 60.8 years. DRE was abnormal in 410 men and was significantly correlated with the age of the patient and tPSA (p<0.001). The tPSA ranged from 0 to 438.3ng/ml with a median, mean and 95th percentile of 1.5, 2.5 and 10.0ng/ml respectively. 341 out of the 438(78%) men selected were subjected to TRBP. Forty-three men had histological diagnosis of CaP, giving an estimated prevalence rate of at least 1.046% or 1046 per 100,000 men of age ≥40. Only 11 (26%) had organ-confined disease while 17 (40%) had locally advanced disease and 15 (35%) men had metastatic disease. The majority of the men, 32 (74%) were reported to have Gleason's score of ≥7.

**Conclusion:**

The prevalence rate of CaP among men aged ≥40 years in Lagos is higher than previously reported in hospital-based study. Majority have advanced and high-grade disease.

## Introduction

There are contradictory guidelines by medical organizations on screening for prostate cancer. The recommendation for prostate cancer screening by the American Urological Association and the American Cancer Society is for all men aged 50 years and above with life expectancy >10 years and starting at age 40-45 years for high-risked men (eg African Americans and those with affected first degree relatives) [1, 2]. However, the National cancer Institute [3] and the United States Preventive Service Task Force [4] do not recommend screening for prostate cancer in the general population or high risk individuals.

The objective of prostate cancer screening is to decrease morbidity or mortality from the disease. However, there is currently no conclusively acceptable evidence to establish whether screening for prostate cancer actually achieves this objective. Prostate cancer screening may reduce mortality from the disease almost by half but with a substantial risk of overdiagnosis [5]. The European Randomized Study of Screening for Prostate Cancer (ERSPC) also demonstrated that population-based screening of men aged 55-75 years can reduce prostate cancer mortality [6, 7] Although population-based screening has not been embraced at the moment, individual patient testing or annual PSA testing is supported. There is therefore no doubt that men should be given individual opportunities take informed decisions whether or not to undertake prostate cancer screening.

Prostate cancer is a leading cancer diagnosis and cause of cancer-related deaths among men. It is the most commonly diagnosed cancer among Nigerian men [8, 9]. An estimated hospital prevalence of 127 per 100,000 in Lagos, Nigeria was reported in 1997 [10]. A recently published data from southwestern Nigeria also reported a hospital prevalence rate of 182.5 per 100,000 male admission in the hospital [11]. However, the true prevalence in the Nigerian community is not known. In the United States, prostate cancer has been described to be more prevalent among the African-Americans. The incidence of prostate cancer among White American men is 156.7 per 100,000 population compared with 248.5 for Black Americans [12]. However, the incidence among the black African community may be underestimated [13, 14]. In this study, we aim to describe the prevalence and the characteristics of prostate cancer among the participants of a community-based prostate cancer awareness program that took place in Lagos, Nigeria with a view to throw more lights into the status of the disease in the community.

## Methods

A prostate cancer awareness program supported by the Lagos State Ministry of Health was carried out in Lagos State, Nigeria. Local Government Areas (LGA) were selected from the 20 LGA in the state for the program which took place in 13 LGA in four batches. Participants were recruited through public service announcements, radio and television jingles and flyers in each of the 13LGA. Men aged ≥40 years were eligible for the exercise.

At the various venues in the LGA, the participants had serum total PSA test and digital rectal examination (DRE).Serum total PSA tests were carried out on blood sample drawn before DRE and all measurements carried out in a single laboratory of the General Hospital, Lagos using chemi-luminescence immunoassay (Beckman Coulter Access 2).The DRE were carried out by trained professionals. Abnormal DRE, suspicious of prostate cancer, was defined as present when the prostate showed any one or more the following features; areas of hardness, nodules, surface irregularity and asymmetry of the prostatic lobes.

The serum PSA results from all the 13 LGA were evaluated together and the mean, median, 95th percentile values were determined. Invitations for transrectal prostate biopsy were based on the serum PSA and the DRE status. Men with serum PSA ≤4.0ng/dL with abnormal DRE, men with serum PSA of >4.0 - 10.0ng/dL with abnormal DRE and all men with serum PSA >10.0ng/dL (which was the calculated 95th percentile serum PSA) irrespective of the DRE status were subjected to transrectal prostate biopsy. Typically, twelve cores of biopsies were systemically obtained and additional samples were obtained from suspicious areas. All specimens were subjected to histopathological examinations.

The data obtained was analysed using SPSS 19.0 for Windows statistical software. Pearson's and Spearman's correlations were used to assess parametric and non-parametric data respectively and p values ≤0.05 were considered significant. The age of the participants were categorized as 40-49, 50-59, 60-69 and ≥70 years.

## Results

A total of 4172men participated inthe awareness program in the13 LGA. The mean and median ages were 60.8 and 60 years respectively. Data for serum PSA and DRE was unavailable for 13 (0.3%) and 25 (0.6%) men who refused either of the procedures respectively. In addition, 24 (0.6%) men who were already on medical treatment or urinary catheter for lower urinary tract symptoms (LUTS) were excluded. Therefore 4110 (98.5%) men were included in the final analysis. Table 1 summarizes the data from the men who participated in the program.

**Table 1:** Serum PSA, DRE status and prevalence of prostate cancer among the participants of the programme classified according to the age group

Parameters	Age (Years)				
	40-49	50-59	60-69	≥70	ALL
**N (%)**	127 (3)	1827 (44)	1419 (35)	737 (18)	4110 (100)
Mean PSA (ng/dL)	1.3	2.0	2.7	3.6	2.5
Median PSA (95^th^ percentile)	0.8 (4.5)	1.3 (6.0)	1.6 (10.1)	1.9 (13.4)	1.5 (10.0)
**N (%) of men with PSA**					
≤4.0	119 (94)	1630 (89)	1118 (79)	531 (72)	3398 (82)
>4.0 – 10.0	7 (5)	121 (7)	172 (12)	114 (16)	414 (10)
>10.0 – 20.0	0 (0)	43 (2)	69 (5)	82 (11)	194 (5)
>20	1 (1)	33 (2)	60 (4)	10 (1)	104 (3)
**DRE Status N (%)**					
Normal	124 (98)	1690 (93)	1250 (88)	636 (86)	3700 (90)
Abnormal	3 (2)	137 (7)	169 (12)	101 (14)	410 (10)
Referred for biopsy N (%)	3 (2)	145 (8)	180 (13)	110 (15)	438 (11)
Biopsy performed N(%)	2 (67)	118 (81)	126 (70)	95 (86)	341 (78)
Prevalence CaProstate N (%)	1 (0.79)	5(0.27)	26(1.83)	11(1.49)	43(1.05)

The serum PSA values ranged from 0 to 438.3ng/ml with median value of 1.5ng/ml and mean of 2.5ng/ml. The 95^th^ percentile PSA value was 10ng/ml. PSA values ≤4.0ng/ml were seen 3398 (82%) men, values >4.0 to ≤10 ng/ml were seen in 414 (10%) men, values >10ng/ml to ≤20ng/ml were seen in 194 (5%) men and values >20ng/ml were seen 104 (3%) men. There was a significant correlation between the serum total PSA and the age of the men (p<0.001). Categorizing the age as 40-49, 50-59, 60-69 and ≥70 years, the median (and the 95th percentile) PSA were 0.8 (4.5), 1.3 (6.0), 1.6 (10.1) and 1.9 (13.4) ng/ml respectively (Table 1).

There were 410(10%) men who had abnormal DRE and the distribution according to the age group is shown in Table 1. The likelihood of finding a patient with abnormal DRE is strongly related with the age of the patient (p<0.001) and the serum total PSA (p<0.001). 270(66%)men with abnormal DRE also had serum total PSA values greater the 95th percentile (10ng/ml) of the population. Forty-four (13.7%) men with abnormal DRE have PSA values greater than 4.0ng/ml but less than 10ng/ml.Only 3 (0.7%) men with PSA value ≤4.0ng/ml had abnormal DRE.

Based on the serum PSA and DRE status, 438 (11%) men were selected for transrectal prostate biopsy. Of these, 44 (10%) men were based on abnormal DRE alone, 124(28%) based men on PSA >95^th^ percentile value (10.0ng/ml) alone and 270 (62%) men based on both the 95^th^ percentile serum PSA and abnormal DRE status. Transrectal prostate biopsies were actually performed on341men (78% of those in whom biopsy was indicated) who consented to it.

Of the 341 men who underwent biopsy, histopathological examination showed that benign nodular hyperplasia (BPH) was found in 242(71.0%), BPH with inflammation in 40 (11.7%), and normal prostate in 16 (4.7%) men. Histopathological diagnosis of prostate cancer was made in 43(12.6%) men. Therefore, the estimated prevalence rate of prostate cancer in the entire cohort of men was 1.046% or 1046per 100,000 men.

The clinical and pathological characteristics of the 43 men with prostate cancer are presented in Table 2. All had adenocarcinoma of the prostate. The serum total PSA were>10ng/L in 41 (95%) men and >4ng/L to ≤10.0ng/L in remaining 2 (5%) men. Of the 43 men with prostate cancer, 39 (91%) had abnormal DRE and the remaining 4 (9%) had normal DRE. Further evaluation of the men with prostate cancer showed that 11 (26%) had organ-confined disease while 17 (39%) men had locally advanced disease and 15 (35%) men had the disease already metastatic to bones (lumbosacral vertebrae and pelvic bones). The majority, 32 (74.4%), of the men were reported to have Gleason's score of 7 and above.

**Table 2:** Characteristics of men with prostate cancer

Variable	N	(%)
**Total**	43	100
**Age**		
40-49	1	2.3
50-59	10	23.3
60-69	24	55.8
≥70	8	18.6
**DRE status**		
Positive	39	90.7
Negative	4	9.3
**Serum PSA (ng/ml)**		
≤4.0	0	0.0
>4.0 – 10.0	2	4.7
>10.0 – 20.0	17	39.5
>20.0	24	55.8
**Gleason's score**		
≤6	11	25.6
7	20	46.5
8-10	12	27.9
**Stage of disease**		
Organ confined	11	25.6
Locally advanced	17	39.5
Metastatic	15	34.9

## Discussion

Prostate cancer screening is not a common practice in Nigeria in spite of prostate cancer being the most commonly diagnosed cancer in Nigerian men [8]. Awareness about prostate cancer is also poor [15, 16]. Majority of our patients therefore usually present in the hospital with the disease in the advanced stage [11]. This community-based testing for prostate cancer which was entirely at no financial cost for the participants has given the opportunity to create more awareness about the disease in the community. It also helped to show the status of the disease in the community better than a hospital-based study.

The estimated prevalence of prostate cancer in this cohort was at least 1046 per 100,000 men of age 40 years and above. This value is much greater than that previously reported in a hospital-based study in Lagos [10] and appears similar to a report from Saudi Arabia [17]. The prevalence rate is expected to be higher than what has been obtained considering that only 78% of men in whom biopsy was indicated actually had biopsy performed and that biopsy was not performed on all men with PSA >4ng/dl. Although the men in this study are community-dwelling, it may be possible that men who have worries about their health are more likely to present. What is of more concern however is the number of supposedly healthy men in the community who have advanced and high grade disease and have not even sought for medical treatment of their condition. Most men with prostate cancer in the cohort (74%) already have advanced disease. This is in agreement with findings of Badmus et al in a hospital-based study from the same region of the country [11]. This is a very high figure when compared to only 4% of prostate cancer patients with metastatic disease at the time of diagnosis in United States where PSA testing is a common practice [18]. In addition, we also had 74% of prostate cancers with high grade disease based on the Gleason's score.

Widespread use of PSA testing may lead to the diagnosis of clinically insignificant tumours and potential overtreatment leading to severe morbidity and unnecessary healthcare cost. Performing transrectal prostate biopsy for all the men with serum total PSA of 4 - 10ng/L could have resulted in a few more cases of prostate cancer identified and therefore a higher value of estimated prevalence rate. However, this would have led to a lot of unnecessary biopsy procedures for the majority of the men.

The normal range of serum total PSA values in our community is generally not known. Values ≤4ng/L are generally used as the normal range. There are suggestions that African men may have a higher PSA value than what are generally accepted values for Caucasian possibly because of a bigger prostatic volume or chronic prostatic inflammation [19]. In this study, the 95^th^ percentile PSA value was 4.5ng/L and 13.4ng/L for men aged 40-49 years and 70 years respectively, while the overall 95^th^ percentile PSA value was 10ng/L. This may suggest that men in our environment generally have a higher PSA value than their Caucasian counterpart.

## Conclusion

This study has demonstrated that there is a high prevalence of prostate cancer in the community in Lagos, much higher than the previously known. The majority of the men already have advanced and high grade disease and have not even sought for medical treatment.
